# Fast oxygen diffusion and iodide defects mediate oxygen-induced degradation of perovskite solar cells

**DOI:** 10.1038/ncomms15218

**Published:** 2017-05-11

**Authors:** Nicholas Aristidou, Christopher Eames, Irene Sanchez-Molina, Xiangnan Bu, Jan Kosco, M. Saiful Islam, Saif A. Haque

**Affiliations:** 1 Department of Chemistry and Centre for Plastic Electronics, Imperial College London, South Kensington Campus, London SW7 2AZ, U.K; 2 Department of Chemistry, University of Bath, Bath BA2 7AY, UK

## Abstract

Methylammonium lead halide perovskites are attracting intense interest as promising materials for next-generation solar cells, but serious issues related to long-term stability need to be addressed. Perovskite films based on CH_3_NH_3_PbI_3_ undergo rapid degradation when exposed to oxygen and light. Here, we report mechanistic insights into this oxygen-induced photodegradation from a range of experimental and computational techniques. We find fast oxygen diffusion into CH_3_NH_3_PbI_3_ films is accompanied by photo-induced formation of highly reactive superoxide species. Perovskite films composed of small crystallites show higher yields of superoxide and lower stability. *Ab initio* simulations indicate that iodide vacancies are the preferred sites in mediating the photo-induced formation of superoxide species from oxygen. Thin-film passivation with iodide salts is shown to enhance film and device stability. The understanding of degradation phenomena gained from this study is important for the future design and optimization of stable perovskite solar cells.

## Abstract (web summary)

Determining the degradation pathways in lead halide perovskites is key to its development as a viable solar technology. Aristidou *et al*. identify iodide vacancies as the preferred sites in mediating the photo-induced formation of superoxide species from intercalated oxygen.

Methylammonium lead halide perovskites have become one of the most promising classes of materials for the development of low-cost, solution-processed optoelectronics such as solar cells, light-emitting diodes and lasers^1^,^2^,^3^,^4^,^5^. Solar cell applications in particular have attracted intense interest in recent years with a rapid rise in power conversion efficiencies of up to 22% for perovskite photovoltaics^6^,^7^,^8^,^9^,^10^,^11^,^12^,^13^,^14^,^15^,^16^,^17^.

However, despite such remarkable progress, serious issues related to the long-term stability of perovskite halides need to be addressed before they can be used successfully in commercial solar cell applications. It has been generally observed that moisture, elevated temperature, oxygen and UV radiation all cause degradation of hybrid perovskite materials and device instability at higher rates than those typically observed in polymer and dye-sensitized photovoltaics^18^,^19^,^20^,^21^,^22^,^23^,^24^,^25^,^26^,^27^,^28^,^29^,^30^,^31^,^32^,^33^,^34^,^35^,^36^,^37^. We recently demonstrated^38^,^39^ that exposure of CH_3_NH_3_PbI_3_ photoactive layers to light and oxygen leads to the formation of superoxide (O_2_^−^) species. This reactive O_2_^−^ species can deprotonate the methylammonium cation (CH_3_NH_3_^+^) of photo-excited CH_3_NH_3_PbI_3_*, leading to the formation of PbI_2_, water, methylamine and iodine,^38^,^39^ as shown schematically in Fig. 1.

This oxygen-induced degradation pathway has been shown to affect the stability of both CH_3_NH_3_PbI_3_ photoactive layers and solar cell devices^40^. Transient absorption spectroscopy studies of interfacial charge transfer in CH_3_NH_3_PbI_3_-based films revealed that such oxygen-induced degradation results in a large decrease in the yield of photo-induced charge carriers^39^. While recent work has demonstrated the importance of electron extraction in reducing the severity of this degradation pathway^20^,^38^,^39^,^40^, it is highly unlikely that charge extraction alone will completely solve this problem. Hybrid lead halide layers deposited from solution typically produce polycrystalline films with varied microstructure and particle morphologies. The impact of film microstructure on charge carrier transport, photoluminescence, device performance parameters (for example, *J*_sc_, *V*_oc_, fill factor) and tolerance to moisture has been reported recently^41^,^42^,^43^,^44^,^45^,^46^,^47^,^48^,^49^,^50^,^51^. It is reasonable to suppose that the particle size and defect chemistry of the films may also influence oxygen diffusion into the perovskite layer and its susceptibility to oxidative reactions.

Studies on CH_3_NH_3_PbI_3_ suggest a significant equilibrium defect concentration of I^−^, Pb^2+^ and CH_3_NH_3_^+^ vacancies at room temperature, which could provide vacancy-mediated pathways for ion transport^52^. Our previous work^53^ and other studies^54^,^55^,^56^,^57^,^58^ indicate that these hybrid perovskites are mixed ionic-electronic conductors, and also implicate vacancy-mediated iodide ion diffusion as being responsible for the observed hysteresis effects. Recent studies have also investigated the role of ion migration in perovskite degradation^21^. Ionic defect and transport phenomena in hybrid perovskites thus have important implications in terms of the long-term stability and performance of perovskite solar cell devices. However, the exact mechanism of oxygen diffusion and the defect species associated with oxygen and light-induced degradation are poorly understood. Additionally, these stability issues raise important questions that have not been fully addressed. Specifically, these questions relate to: (i) the origin of the observed fast rate of oxygen-induced degradation of CH_3_NH_3_PbI_3_ films and (ii) the relationship between the film particle size, oxygen transport, intrinsic vacancies and the mechanism of oxygen-induced degradation.

Here we combine experimental and computational methods to address these important questions at the microscopic level, extending our previous work on hybrid perovskites^38^,^39^,^40^,^53^. Specifically, we have probed the dynamics of oxygen diffusion in perovskite films and investigated how the film morphology influences reactivity to molecular oxygen. Oxygen diffusion into perovskite films is observed to occur remarkably fast with, for example, a typical film (500 nm thick) reaching complete saturation within 10 min. Density functional theory (DFT) calculations also reveal the importance of iodide vacancies as reaction mediators to form superoxide species. The results provide valuable insights for the design of perovskite devices exhibiting improved environmental stability.

## Results

### Oxygen diffusion

We first consider the dynamics of oxygen diffusion in hybrid perovskite films. CH_3_NH_3_PbI_3_ and CH_3_NH_3_PbI_3_(Cl) were chosen for a comparative study, since both have been widely tested in photovoltaic devices. (Note that the formula CH_3_NH_3_PbI_3_(Cl) refers to a perovskite material fabricated from a combination of iodide and chloride precursors.) Of the two systems, CH_3_NH_3_PbI_3_(Cl) films are reported to be more stable^59^,^60^ but the origin(s) of this superior stability remains unclear. In our study, films were fabricated as described in the Methods section onto clean glass substrates. X-ray diffraction (XRD) (see Supplementary Fig. 1) was used to characterize both materials, and confirmed that in both cases CH_3_NH_3_PbI_3_ is the compound formed. We recognize that the concentration of chlorine within the CH_3_NH_3_PbI_3_(Cl) film may be too low to determine through XRD. The presence of any chlorine may give rise to differences in charge carrier recombination, which could influence stability, but the low Cl levels mean this is unlikely to be the dominant effect. Chlorine substitution is thus unlikely to be responsible for the improved stability of the material.

Isothermal gravimetric analysis (IGA) was used to probe the dynamics of oxygen diffusion as illustrated in Fig. 2a. In a typical experiment, the sample chamber with the perovskite film was first flushed with He gas for 30 min. Next, the weight of the perovskite sample was recorded as a function of exposure to dry air (N_2_=80%, O_2_=20%) over the course of 20 min at room temperature (25 °C). As can be seen from the IGA traces in Fig. 2a, oxygen diffusion into both CH_3_NH_3_PbI_3_ and CH_3_NH_3_PbI_3_(Cl) films was rapid, with saturation being achieved within 5–10 min. Further evidence for the fast rate of oxygen diffusion in these two materials was obtained from time-of-flight secondary ion mass spectrometry (ToF-SIMS) measurements, in which the depth profile of oxygen throughout the films was determined. In these experiments, films of both CH_3_NH_3_PbI_3_ and CH_3_NH_3_PbI_3_(Cl) were soaked in dry air in the absence of light and ToF-SIMS data collected. The ToF-SIMS images shown in Fig. 2b,c represent slices through CH_3_NH_3_PbI_3_ and CH_3_NH_3_PbI_3_(Cl) layers at a depth of approximately 200 nm in the films, after soaking for 30 min. 3D profiles (raw data) showing oxygen concentration as a function of film depth are also provided in Supplementary Fig. 2. Taken together, these findings demonstrate that oxygen enters the perovskite samples and is uniformly distributed throughout the films. As the time period for oxygen uptake to reach saturation is in agreement with the fast degradation rate previously observed in CH_3_NH_3_PbI_3_ photoactive layers and devices^38^,^39^,^40^, we conclude that the high sensitivity of CH_3_NH_3_PbI_3_ devices to oxygen is owed, in part, to the rapid rate at which oxygen can diffuse into the films.

From the IGA data we can estimate the air (80% N_2_ and 20% O_2_) diffusion coefficient, *D*_a_, to be in the range 10^−7^–10^−9^ cm^2^ s^−1^ for the CH_3_NH_3_PbI_3_ perovskite material (calculations provided in SI). This value is comparable to the fast diffusion of gases into polymer thin films where, for example, the diffusion coefficient^61^ in poly-(3-hexylthiophene-2,5-diyl) (P3HT) thin films is of the order of 10^−8^ cm^2^ s^−1^. It is widely accepted that these fast diffusion kinetics are responsible for the relatively low stability of semiconducting polymer films to molecular oxygen^62^,^63^,^64^. As such, we propose that the fast oxygen diffusion kinetics are critical to the observed oxygen- and light-induced degradation rates seen in perovskite-based optoelectronic devices.

### Particle size dependence

Next we considered the effect of visible light and oxygen on the relative stability of CH_3_NH_3_PbI_3_ and CH_3_NH_3_PbI_3_(Cl) films and devices. Figure 3a,b shows the absorption spectra of CH_3_NH_3_PbI_3_ and CH_3_NH_3_PbI_3_(Cl) films on glass substrates measured as a function of ageing under illumination in dry air. Figure 3a,b reveals that both perovskite materials rapidly degrade under these conditions. However, it is apparent that CH_3_NH_3_PbI_3_(Cl) films degrade at a significantly slower rate than CH_3_NH_3_PbI_3_ films. Figure 3c shows the power conversion efficiency (PCE) versus time profile for CH_3_NH_3_PbI_3_ and CH_3_NH_3_PbI_3_(Cl)-based solar cells, with the devices being continuously aged in dry air under light. The corresponding current–voltage curves are provided as Supplementary Fig. 3 in Supplementary information. For these studies, a solar cell architecture of the type [FTO/compact-TiO_2_/mesoporous-TiO_2_/perovskite/spiro-OMeTAD/Au] was employed and all PCE measurements were performed on un-encapsulated devices as in previous work^44^. The PCEs of the solar cells were determined from current–voltage characteristics ascertained at regular time intervals over the course of 4 h. In Fig. 3c, CH_3_NH_3_PbI_3_(Cl) devices show a relatively small 10% drop in PCE over the 4-h ageing period whereas the CH_3_NH_3_PbI_3_ devices display a more substantial (≈80%) drop in PCE under the same ageing conditions. The better stability of the CH_3_NH_3_PbI_3_(Cl) solar cells (compared to CH_3_NH_3_PbI_3_) is consistent with the absorption data in Fig. 3a,b, as well as other recent reports^59^. The difference in stability between CH_3_NH_3_PbI_3_ and CH_3_NH_3_PbI_3_(Cl) observed here may be related to the smaller size of the particles.

The next question that arises relates to the origin of the difference in stability observed between CH_3_NH_3_PbI_3_ and CH_3_NH_3_PbI_3_(Cl). To identify the cause(s) for this difference we first determined the yield of photo-induced superoxide (O_2_^−^) formation for the two perovskites. We have previously established that O_2_^−^ is the key reactive species responsible for the hybrid perovskite and device degradation^38^,^40^. Here the yield of O_2_^−^ was determined for both CH_3_NH_3_PbI_3_ and CH_3_NH_3_PbI_3_(Cl) films using a hydroethidine (HE) fluorescent probe (as described in the Methods section). As detailed elsewhere^38^, the degradation effect we observe cannot be ascribed to degradation of the hole transporting material spiro-OMeTAD since we have not used spiro-OMeTAD in these experiments.

Figure 3d shows the rate of increase in HE emission and, accordingly, the O_2_^−^ generation yield. The data in Fig. 3d show that CH_3_NH_3_PbI_3_ produces a significantly higher yield of superoxide than CH_3_NH_3_PbI_3_(Cl), consistent with the film and device stability data in Fig. 3a–c. It is reasonable to propose that this difference in reactivity stems from differences in the particle sizes within the films. For example, the presence of chloride ions in the precursor mixture is known to slow down the rate of crystal formation, leading to larger crystals^28^,^29^. This was confirmed by taking scanning electron microscopy (SEM) images of the CH_3_NH_3_PbI_3_ and CH_3_NH_3_PbI_3_(Cl) films. Figure 3e,f shows that the CH_3_NH_3_PbI_3_(Cl) film consists of crystal domains that are several hundreds of nanometres in diameter, considerably larger than those observed in the CH_3_NH_3_PbI_3_ sample. This strongly suggests that crystal size plays a crucial role in determining the stability.

In order to test this hypothesis, we next investigated, in a systematic manner, the stability of CH_3_NH_3_PbI_3_ films composed of different crystal sizes. The controlled growth of CH_3_NH_3_PbI_3_ crystals was achieved following the method previously reported by Grätzel and co-workers^50^. In this way, we prepared samples with small (100 nm), medium (160 nm) and large (250 nm) perovskite crystals as confirmed by SEM. Representative SEM images are shown in Fig. 4a–c. In Fig. 4d the magnitude of the absorbance at 750 nm is plotted against ageing time, with black, red and blue curves corresponding to films composed of small (sample 1), medium (sample 2) and large (sample 3) CH_3_NH_3_PbI_3_ crystallites, respectively. The corresponding absorption spectra are provided in Supplementary Fig. 4. We stress that these measurements were recorded until the CH_3_NH_3_PbI_3_ film had turned completely yellow, indicating full conversion of the perovskite to the degradation product, PbI_2_. It is apparent from the data in Fig. 4d that the films with large CH_3_NH_3_PbI_3_ crystals are considerably more stable than the films composed of small CH_3_NH_3_PbI_3_ crystals. We note that all three samples showed similar rates of oxygen uptake, with the films reaching saturation within 10 min, as determined by IGA experiments (Supplementary Fig. 5).

Next, we consider the possible correlation between CH_3_NH_3_PbI_3_ crystal size, materials stability and superoxide (O_2_^−^) generation yield. Figure 4e shows the measured rate of increase of O_2_^−^ species in these three samples; a correlation is observed between the CH_3_NH_3_PbI_3_ crystallite size, stability (that is, tolerance to visible light and oxygen stress) and O_2_^−^ yield. It is evident that the CH_3_NH_3_PbI_3_ films with large crystallites show a relatively low yield of superoxide formation and display better stability. Conversely, the CH_3_NH_3_PbI_3_ films composed of smaller crystallites show a higher yield of superoxide generation and rapidly degrade within 2 days. There thus appears to be a direct correlation between yield of superoxide generation (and subsequently degradation rate) and perovskite crystallite size.

### Superoxide formation sites

To obtain atomic-scale insights into the energetics and defect mechanisms of the degradation process we have used *ab initio* simulations based on DFT. We first probed the reaction with oxygen (with no photo-generated electrons in the perovskite lattice), described by the following equation:


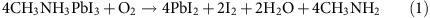


The enthalpy was calculated to be +1.60 eV per O_2_ molecule, indicating that the reaction in the absence of light is unfavourable, which is consistent with observation. We have already shown that in order for the overall degradation reaction to occur, the film must be exposed to both O_2_ and light in order for superoxide species to form^38^,^39^. The implication is that O_2_ acts as an electron scavenger and absorbs electrons generated by light or an external electrical bias.

**Figure 1:**
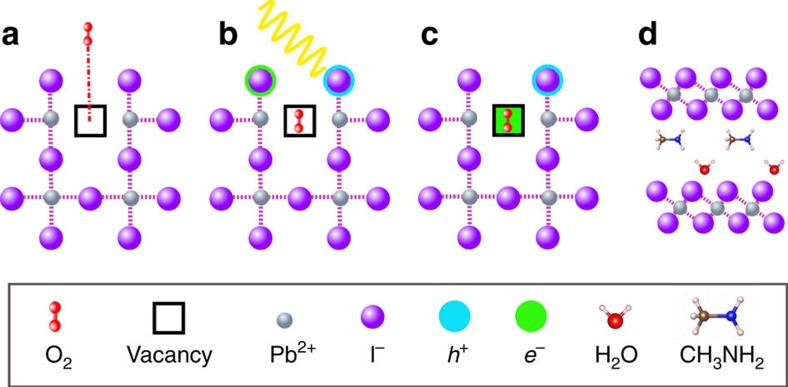
Oxygen-induced photo-degradation. Schematic representation of the reaction steps of O_2_ with CH_3_NH_3_PbI_3_. (**a**) Oxygen diffusion and incorporation into the lattice, (**b**) photoexcitation of CH_3_NH_3_PbI_3_ to create electrons and holes (**c**) superoxide formation from O_2_, and (**d**) reaction and degradation to layered PbI_2_, H_2_O, I_2_ and CH_3_NH_2_.

**Figure 2:**
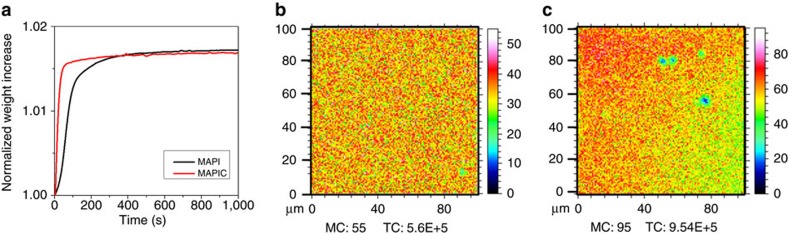
Oxygen diffusion. (**a**) Isothermal gravimetric analysis plot (IGA) of CH_3_NH_3_PbI_3_ (MAPI) and CH_3_NH_3_PbI_3_(Cl) (MAPIC) thin films coated on non-conductive cleaned glass with 30 min of Helium soaking before oxygen exposure, where *t*=0 corresponds to the time at which oxygen was introduced into the system. (**b**,**c**) ToF-SIMS surface images (100 × 100 μm) of CH_3_NH_3_PbI_3_ and CH_3_NH_3_PbI_3_(Cl) respectively after exposure to dry air flow with no illumination, where the maximum counts (MC) and the total number of secondary oxygen ion counts (TC) are shown with the colour scales corresponding to the interval [0, MC].

The next step in the reaction sequence is for superoxide O_2_^−^ to react with photo-oxidized CH_3_NH_3_PbI_3_* to produce PbI_2_, I_2_, H_2_O and CH_3_NH_2_ according to the following reaction (illustrated in Fig. 1):


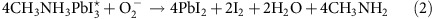


In contrast to reaction (1), the calculated enthalpy for reaction (2) is negative (−1.40 eV per O_2_ molecule), indicating that the degradation reaction is now highly favourable, again in agreement with observation. This indicates that both the photo-oxidized CH_3_NH_3_PbI_3_ and the O_2_^−^ species are unstable with respect to the reaction products.

A key step in the degradation reaction is the formation of the superoxide species according to O_2_+*e*^−^=O_2_^−^. This raises the important question of where the superoxide forms in the crystal lattice. It is plausible that ionic defects such as iodide vacancies play a key role in mediating superoxide formation from O_2_ and hence the degradation reaction. As noted, recent work^52^ indicates a significant population of intrinsic Schottky-type defects (a stoichiometric combination of anion and cation vacancies), whose formation can be expressed using Kröger–Vink notation as:


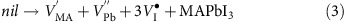


where *nil* represents the perfect (defect-free) CH_3_NH_3_PbI_3_ lattice, *V* indicates a vacancy, subscripts indicate the type of lattice site and superscripts the effective charge of the defect (a dot for each positive charge and prime for each negative charge). Such vacancy defects may act as molecular or charge traps which in turn could mediate the electron transfer reaction with oxygen. It is known that the anion vacancy in binary lead halides can trap a photoelectron to form an F centre^24^.

To investigate this process, we calculated the energetics for superoxide formation from O_2_ molecules on various lattice and vacancy sites in the CH_3_NH_3_PbI_3_ perovskite structure; the most favourable configurations and the corresponding superoxide formation energies are summarized in Fig. 5. Two main findings emerge. First, superoxide formation by direct electron transfer from the perovskite to oxygen is energetically favourable. Analysis of the electron density and bond lengths shows that the photo-generated electron resides on the O_2_ molecule; upon adsorption the O_2_ bond length increases from 1.22 to 1.33 Å and the species becomes spin polarized. Second, superoxide formation energies indicate that vacant iodine sites are the preferred location for the reduction process in the crystal bulk. Interestingly, an iodide ion is of similar size to the superoxide species (as illustrated in Fig. 6), and in occupying an iodide vacancy, the superoxide ion restores the full octahedral coordination of Pb^2+^.

Further evidence for the favourable energetics of O_2_ reduction is given by the calculated band structures for O_2_ incorporation into CH_3_NH_3_PbI_3_ reported in Fig. 7. In Fig. 7a it can be seen that the unoccupied oxygen π* anti-bonding orbital is located in the middle of the CH_3_NH_3_PbI_3_ band gap where it can readily act as an acceptor state for photo-excited electrons in the conduction band. Moreover, when the O_2_^−^ superoxide species occupies an iodide vacancy (Fig. 7b), the oxygen states are shifted down into the valence band as a result of changes in bonding interactions, clearly indicating that it is even more energetically favourable for O_2_ to be reduced by photo-excited electrons in the conduction band.

### Proposed degradation mechanism

**Figure 3:**
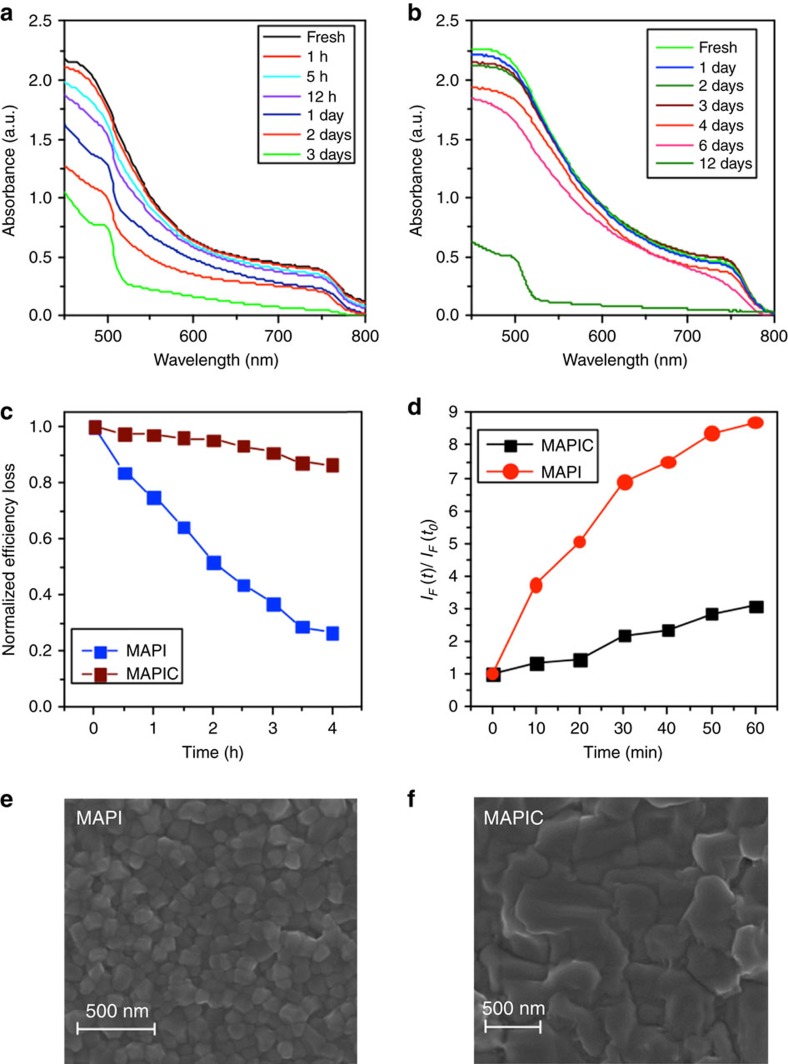
Stability comparison of CH_3_NH_3_PbI_3_ and CH_3_NH_3_PbI_3_(Cl). (**a**,**b**) Light absorption spectrum for ageing CH_3_NH_3_PbI_3_ and CH_3_NH_3_PbI_3_(Cl) under dry air and illumination (25 mW cm^−2^) respectively. (**c**) Normalized power conversion efficiency loss for photovoltaic devices employing CH_3_NH_3_PbI_3_ (referred to as MAPI) and CH_3_NH_3_PbI_3_(Cl) (referred to as MAPIC) as the light harvesting materials in an [FTO/planar-TiO_2_/mesoporous-TiO_2_/perovskite/spiro-OMeTAD/Au] architecture. J-V curves obtained for these studies are shown in Supplementary Fig. 4. (**d**) Normalized fluorescence intensity increase of the HE probe at 610 nm (excitation at 520 nm). *I*_F_(*t*) is the fluorescence maximum at time *t*, while *I*_F_(*t*_0_) is the background fluorescence intensity. *I*_F_(*t*)/*I*_F_(*t*_0_) ratio corresponds to the yield of superoxide generation for the perovskite films. (**e**,**f**) Surface SEM images of CH_3_NH_3_PbI_3_ and CH_3_NH_3_PbI_3_(Cl) films deposited on cleaned glass substrates.

**Figure 4:**
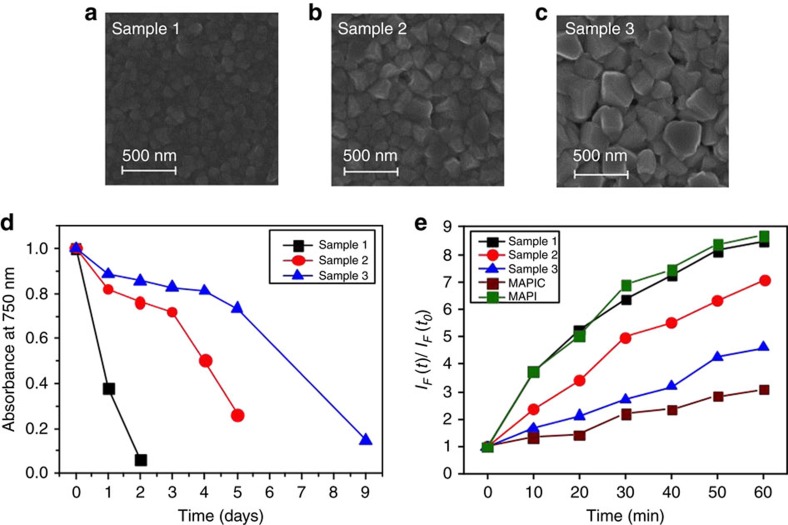
Particle size effects. (**a**–**c**) Surface SEM images of small (sample 1), medium (sample 2) and large (sample 3) crystal sizes of CH_3_NH_3_PbI_3_. (**d**) Normalized absorbance decay at 750 nm for methylammonium lead iodide of sample 1 (small crystals, 100 nm), sample 2 (medium crystals, 150 nm) and sample 3 (large crystals, 250 nm) with degradation conditions of illumination (25 mW cm^−2^) and dry air. (**e**) Superoxide yield plot for CH_3_NH_3_PbI_3_ (with small (black), medium (red), large (blue) crystal sizes and a toluene dripped prepared sample (green) and CH_3_NH_3_PbI_3_(Cl) (brown).

**Figure 5:**
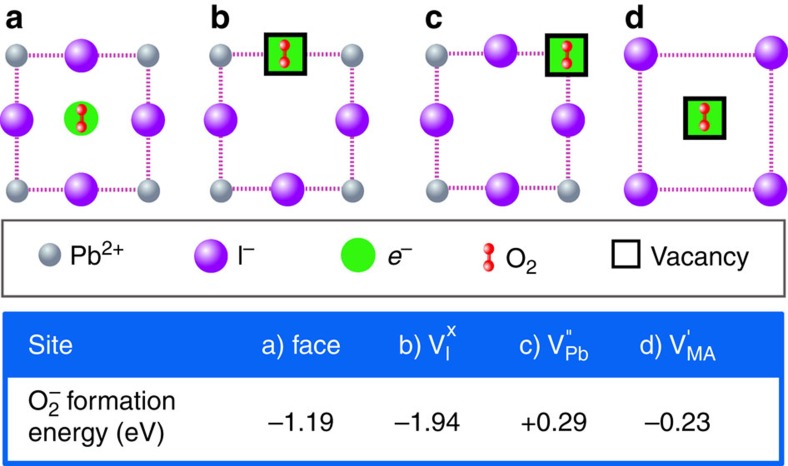
Lattice sites for superoxide formation. Schematic representation of possible O_2_ binding and reduction sites in CH_3_NH_3_PbI_3_ ([001] plane) and corresponding superoxide formation energy: (**a**) face site neighbouring four iodide ions, (**b**) neutral iodide vacancy (with a photoelectron on the defect site) and negatively charged lead (**c**) and methylammonium (**d**) vacancies (with no photoelectron on them since this was found to be unphysical).

**Figure 6:**
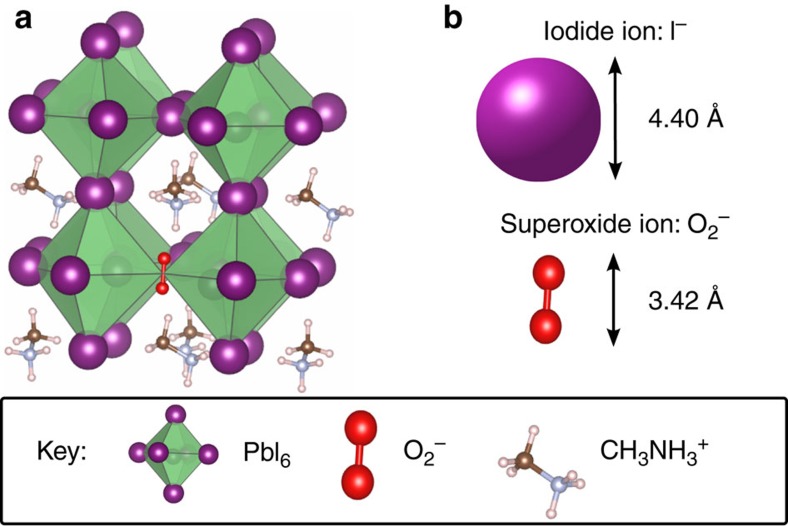
Size comparison of superoxide and iodide anions. Atomic structure of CH_3_NH_3_PbI_3_ showing (**a**) superoxide ion, O_2_^−^, occupying an iodide vacancy, 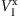 (for clarity, a pseudo-cubic sub-region of the structure is shown and not the full tetragonal supercell used in the calculations). (**b**) Comparison of relative size of iodide and superoxide anions (using ionic radius of I^−^ and, for the superoxide ion, interpolation between covalent radius in O_2_ and ionic radius of O_2_^−^).

**Figure 7:**
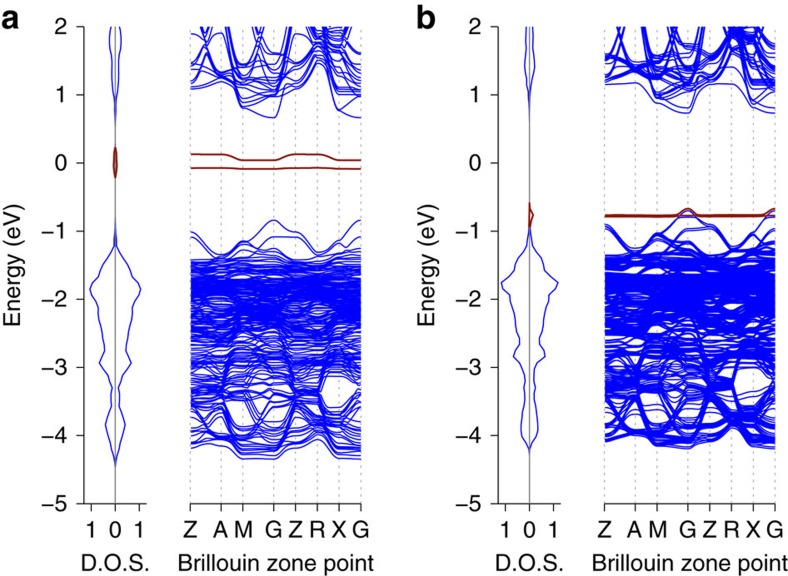
Band structure and density of states for oxygen incorporation into CH_3_NH_3_PbI_3_. (**a**) O_2_ in defect-free CH_3_NH_3_PbI_3_ and (**b**) O_2_ at iodide vacancy in CH_3_NH_3_PbI_3_. Key: blue—CH_3_NH_3_PbI_3_, red—O_2_. Note the band structure is folded due to the use of a large supercell.

To summarize our four key observations: (i) photo-induced oxygen degradation of CH_3_NH_3_PbI_3_ shows a strong particle size dependence, (ii) structural degradation from the black perovskite phase to the yellow lead PbI_2_ phase occurs on a timescale of days (see absorbance data, Fig. 3a), whereas the device performance degrades in a matter of hours (see efficiency data, Fig. 3c), (iii) rapid oxygen uptake occurs on a timescale of less than 1 h and results in superoxide generation, and (iv) superoxide formation is always associated with degradation and is facilitated by iodide vacancies.

**Figure 8:**
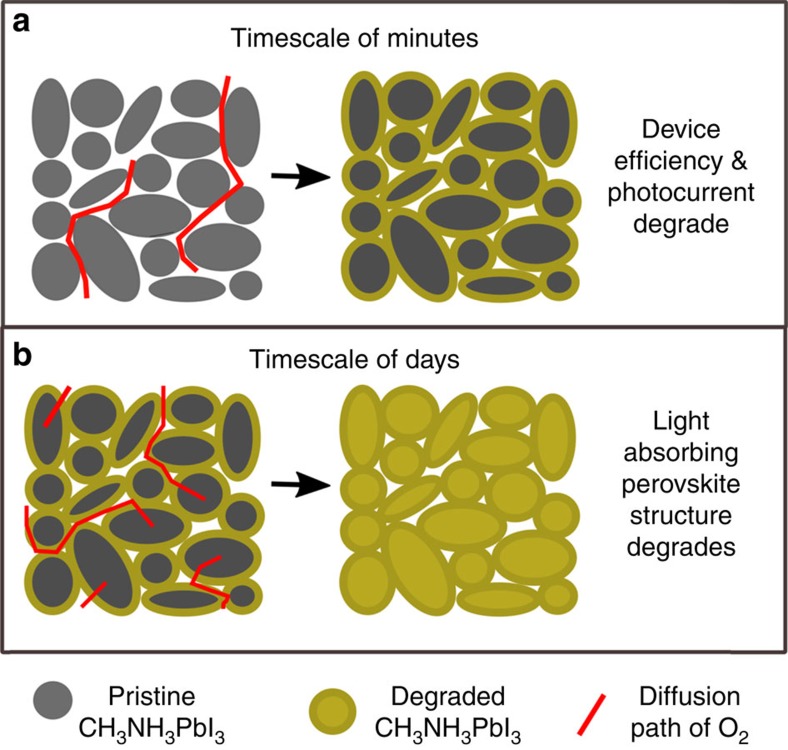
Oxygen diffusion pathways and associated degradation regions. (**a**) O_2_ diffusion in inter-particle regions and initial reaction with particle surfaces over a timescale of hours leading to reduction in device efficiency, open circuit voltage and photocurrent and (**b**) O_2_ diffusion into particle bulk regions over a time scale of days leading to a phase change from the photo-absorbing perovskite phase into the non-absorbing lead iodide phase (yellow).

To rationalize these findings, we propose a mechanism that is summarized schematically in Fig. 8. The CH_3_NH_3_PbI_3_ sample is under illumination throughout this process to provide a constant source of photo-excited electrons in the bulk and surface regions. Oxygen is admitted to the sample and diffuses between the particles and within an hour permeates the inter-particle regions (Fig. 8a). Superoxide species immediately begin to form at the particle surfaces as O_2_ is reduced while occupying surface iodide vacancies. Over the first few hours there is an initial reaction between these superoxide species and the particle surfaces, leading to their passivation. The degradation of the particle surfaces prevents the extraction of the photocurrent^65^, causing the device properties such as efficiency (PCE) to decline rapidly. On a timescale of days (Fig. 8b) oxygen diffuses into the interior of the particles where it occupies bulk iodide vacancies while being reduced, leading to full structural degradation of the material.

For both the surface and bulk reactions we would expect the particle size and iodide vacancy levels to influence the rate of reaction. It is well known that particle surfaces are normally much more reactive than the bulk; in the halide perovskites it has been shown that different particle facets can display markedly different photovoltaic behaviour^66^. Furthermore, Haruyama *et al*.^67^ have shown that in CH_3_NH_3_PbI_3_ the most prominent surfaces are vacancy rich with defect concentrations of 10^12^ cm^−2^, and our data show that oxygen reduction occurs most readily at the iodide vacancies. Thus, the increased initial degradation rate for smaller crystallites may be directly related to the surface vacancy concentration. Figure 3 shows SEM micrographs of large crystallites with typical sizes of ca. 250 nm and small crystallites of ca. 100 nm. Based on these average values, we would expect the number density of surface vacancies to be around 2.4 × 10^17^ cm^−3^ in the large crystallites and around 6.0 × 10^17^ cm^−3^ in the small crystallites. The small crystallites thus provide more than twice as many surface adsorption/reaction sites per unit volume as the large crystallites.

In addition, bulk adsorption/reaction sites will contribute significantly to the rate of degradation over long time-scales. Iodide vacancy concentrations in the bulk have been estimated^52^ at 10^22^ cm^−3^, which suggests that in a typical particle, bulk vacancies will be five orders of magnitude more numerous than surface vacancies. Since our ToF-SIMS data show that O_2_ can diffuse into the bulk, the increased degradation rate for smaller crystallites may be due to the shorter O_2_ diffusion path lengths to the particle interiors. In general, the characteristic time constant, *t*, for ion diffusion is given by *t*=*L*^2^/*D*, where *L* is the diffusion path length and *D* is the diffusion coefficient. The rate of oxygen permeation into the bulk of CH_3_NH_3_PbI_3_ can be expected to drop by a factor of four for every doubling of the particle size. This model can thus account for our observations. We recognize that other factors such as the rate of reaction between the superoxide species and methylammonium cation may also limit the overall rate of degradation, but such topics are left for future investigations.

### Film passivation using salt additives

Finally, a key question that arises relates to whether the stability of CH_3_NH_3_PbI_3_ films can be improved by inhibiting superoxide formation at iodide vacancies, for example, by defect passivation. To investigate this, a solution of either chloride or iodide salts of cations phenylethylammoniun or methylammonium and trimethlsulfonium iodide was spin-coated onto the CH_3_NH_3_PbI_3_ perovskite films. Ionic passivating agents carrying bulky cations were chosen to ensure that substitution with methylammonium cations in the perovskite was unfavourable. The successful preparation of the films was confirmed by XRD analysis (see Supplementary Fig. 6).

**Figure 9:**
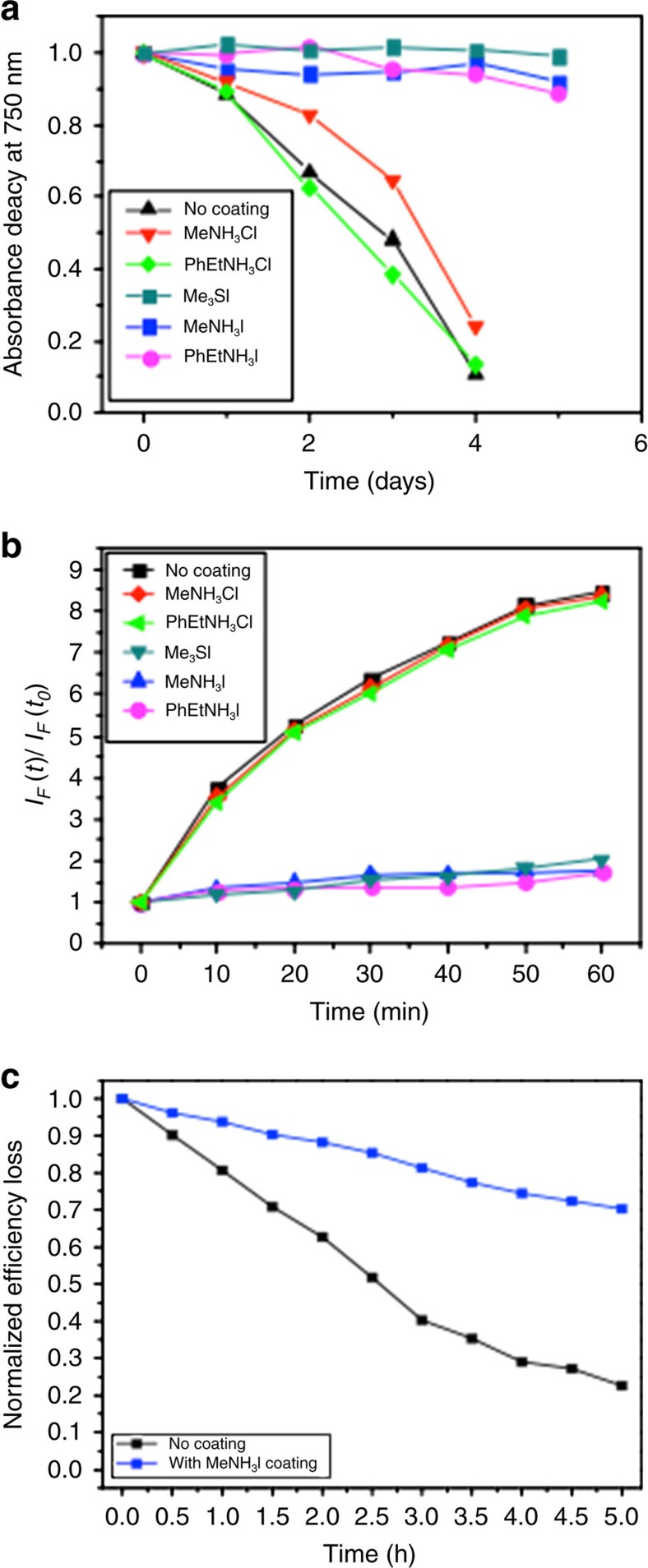
Film passivation using salt additives. (**a**) Normalized absorbance decay at 750 nm under illumination (25 mW cm^−2^) and dry air flow for a pristine CH_3_NH_3_PbI_3_ and CH_3_NH_3_PbI_3_ coated with phenylethylammonium iodide (PhEtNH_3_I), methylammonium iodide (MeNH_3_I), trimethylsulfonium iodide (Me_3_SI), phenylethylammonium chloride (PhEtNH_3_Cl) and methylammonium chloride (MeNH_3_Cl) as described in the experimental section. (**b**) Superoxide yield plot comparing the generation of superoxide for CH_3_NH_3_PbI_3_ treated with the coatings and without. (**c**) Normalized power conversion efficiency loss for photovoltaic devices employing CH_3_NH_3_PbI_3_ with and without a 10 mM treatment of MeNH_3_I as the light harvesting material in an [FTO/planar-TiO_2_/mesoporous-TiO_2_/perovskite/spiro-OMeTAD/Au] architecture. J-V curves obtained for these studies are shown in the Supplementary Fig. 13.

The stabilities of the coated films were examined after exposure to light and oxygen. As can be seen in Fig. 9a (raw absorbance data are presented in Supplementary Fig. 7), all the iodide salt-coated samples display an enhanced tolerance to light and oxygen (relative to the uncoated control sample), showing little or no degradation over the ageing time period. In contrast, the chloride salt derivatives showed no stability enhancement, exhibiting a comparable rate of degradation as that of an uncoated perovskite sample. We note that the iodide treated films showed no sign of any degradation over 3 weeks of ageing under light and oxygen. Furthermore, the enhanced stability of the coated samples is consistent with the superoxide generation data shown in Fig. 9b. In particular, it can be seen again that all the iodide salt coated samples show a significantly lower yield of superoxide species than the uncoated sample and the chloride salt-treated samples.

To confirm that the iodide salt treatments are indeed passivating iodide defects rather than some other effect, we performed time-resolved photoluminescence, IGA, SEM and superoxide yield measurements. IGA data presented in Supplementary Fig. 8 indicate similar fast oxygen diffusion kinetics in both CH_3_NH_3_I treated and untreated films. In contrast, a control sample comprising a CH_3_NH_3_PbI_3_ film encapsulated in glass showed little or no weight increase upon exposure to oxygen; in this instance the glass layer functions as an oxygen blocking layer. In addition, time-resolved photoluminescence measurements were performed on perovskite films treated with different concentrations (0.001 M, 0.005 M and 0.01 M) of CH_3_NH_3_I. As can be seen from the data provided in Supplementary Fig. 9, increasing the iodide salt concentration leads to an increased photoluminescence lifetime. Moreover, this observation is consistent with the iodide salt treatment reducing the number of defects and therefore the number of trap states for non-radiative recombination, resulting in longer emission lifetimes. Recent studies^68^,^69^ have found that alkali metal halide salts introduced at the perovskite interface can decrease halide vacancy levels, resulting in improved device performance.

Next, the relative stability and superoxide yield of CH_3_NH_3_PbI_3_ films treated with different concentrations of CH_3_NH_3_I were investigated. The relationship between CH_3_NH_3_I concentration, stability and superoxide yield is shown in Supplementary Fig. 10, which shows that increasing the concentration of CH_3_NH_3_I salt leads to lower yields of superoxide generation and consequently better stability. Finally, surface SEM images were taken of a treated and an untreated CH_3_NH_3_PbI_3_ film to show that the treatment does not induce any significant morphological changes to the film's surface (provided in Supplementary Fig. 11). Taken together, the results in Supplementary Figs 8–11 strongly suggest that salt treatments passivate the crystal defects rather than providing a physical barrier layer to oxygen diffusion.

Passivation of crystal defects using iodide salts reduces superoxide yields and enhances film stabilities, but it is not known whether they improve device stability. To address this question, cells were fabricated and characterized under different conditions (as described in the experimental section). The results, shown in Fig. 9c and Supplementary Figs 12,13 and Supplementary Table 1, indicate that solar cells that use CH_3_NH_3_I-coated CH_3_NH_3_PbI_3_ films exhibit better stability than those that use uncoated perovskite layers. More specifically, exposure of devices using uncoated perovskite layers to light and dry air for just 2.5 h leads to a 50% drop in the PCE. In contrast, devices containing CH_3_NH_3_I coated layers exhibit a significantly smaller drop in efficiency (10%) over the same ageing period. The relationships between concentration of the CH_3_NH_3_I coating in solution and the device stability are shown in Supplementary Figs 12,13 and Supplementary Table 1. It is clear from these results that increasing the concentration of CH_3_NH_3_I salt leads to progressively higher stability.

These results further confirm that the iodide anion from the salt passivates the iodide vacancies in the crystal, and by occupying the otherwise vacant iodide sites leads to increased stability by suppressing superoxide formation. We note that degradation of the coated devices is not halted completely, which suggests that other factors also influence overall device stability. For example, it is possible that iodide vacancies in the bulk are not filled by iodide ions from the coating and these can still act as sites for superoxide generation. Nevertheless, it is clear from the present findings that the iodide salt coatings lead to significant improvements in device stability.

## Discussion

The present study suggests that iodide defects in the CH_3_NH_3_PbI_3_ structure are key sites for superoxide formation. In addition, the results demonstrate that iodide salt treatment can be employed to reduce the number of problematic iodide vacancies, thereby hindering the electron transfer reaction that generates superoxide species. Although this study is not exhaustive it does highlight a critical issue for further work, and could include probing the combined effect of oxygen and water on CH_3_NH_3_PbI_3_ as well as large-scale atomistic simulations of oxygen diffusion and iodine interstitials.

In conclusion, a combination of IGA, photoluminescence, SIMS and *ab initio* simulation techniques has provided mechanistic insights into oxygen- and light-induced degradation of perovskite solar cells. We found that fast oxygen diffusion into CH_3_NH_3_PbI_3_ films is accompanied by formation of superoxide species, which are critical to oxygen-induced degradation. The yield of superoxide (O_2_^−^) species and thus the degradation rate are dependent on crystallite size when exposed to light and oxygen: perovskite films composed of small crystallites show high yields of photo-induced superoxide formation and therefore low stability. *Ab initio* simulations indicate that iodide vacancies are the preferred sites in mediating the photo-induced formation of superoxide species from O_2_. We also demonstrated that thin-film passivation with iodide salts leads to reduced superoxide formation, and consequently enhanced film and device stabilities. These combined results improve our fundamental understanding of degradation phenomena in perovskite solar cells, and provide a strategy for greatly improving their long-term stability.

## Methods

### Materials and synthesis

All chemicals were purchased from Sigma-Aldrich and used as received, except TiO_2_ nanoparticles from Dyesol and methylammonium iodide (MAI), which were synthesized in the lab. Methylamine 33% wt solution in ethanol (6.2 ml, 0.046 mol) was cooled down in an ice bath. Hydroiodic acid 55% wt solution in water (10 ml, 0.073 mol) was then added dropwise under vigorous stirring. The reaction was stirred for 1 h at 0 °C. The product precipitated from the solution as a white-yellowish solid. Ethanol (5 ml) was added to ensure full precipitation of the solid, which was filtered and washed with cold ethanol. Recrystallization of the product in ethanol/diethyl ether afforded the pure compound as white crystalline solid (6.4 g, 87%). Synthesis of phenyl-ethylammonium iodide (PEAI): Phenylethylamine 33% wt solution in ethanol (6.2 ml, 0.046 mol) was cooled down in an ice bath. Hydroiodic acid 55% wt solution in water (10 ml, 0.073 mol) was then added dropwise under vigorous stirring. The reaction was stirred for 1 h at 0 °C. The product precipitated from the solution as a white-yellowish solid. Ethanol (5 ml) was added to ensure full precipitation of the solid, which was filtered and washed with cold ethanol. Recrystallization of the product in ethanol/diethyl ether afforded the pure compound as white crystalline solid.

### Film fabrication

All films were deposited onto clean glass substrates of ca. 1 cm by 1 cm in size. The glass substrates were washed sequentially in acetone, water and isopropylalcohol (IPA) under sonication for 10 min during each washing cycle. A Laurell Technologies WS-650MZ-23NPP Spin Coater was used to fabricate the films. (a) CH_3_NH_3_PbI_3_: A 1 M solution of CH_3_NH_3_PbI_3_ was formed by adding PbI_2_ in a 1:1 molar ratio with MAI in a solvent mixture of 7:3 γ-butyrolactone to DMSO. This solution was then spin-coated onto the substrates using a consecutive two-step spin program under a nitrogen atmosphere in a glove box. The first spinning cycle was performed at 1,000 r.p.m. for 10 s followed by 5,000 r.p.m. for 20 s, as reported by Jeon *et al*.^70^. During the second phase, the substrate was treated with toluene (ca. 350 ml) drop-casting. The films were then annealed at 100 °C for 10 min. (b) Crystal size variation: Films were prepared with controlled crystal size in accordance with the two-step deposition method described by Grätzel *et al*.^50^. Twenty microlitres of a 1 M solution of PbI_2_ in DMF was spin-coated onto glass substrates at 3,000 r.p.m. for 5 s and then at 6,000 r.p.m. for another 5 s. The films were then annealed at 40 °C for 3 min followed by heating at 100 °C for 5 min. Once the films had cooled to room temperature, 200 μl of 0.038 M (sample 3), 0.050 M (sample 2) and 0.063 M (sample 1) MAI in IPA solution were loaded on top of them for 20 s, before spinning at 4,000 r.p.m. for 20 s. Films were then annealed at 100 °C for 5 min. (c) Chlorine treated CH_3_NH_3_PbI_3_: A 1 M solution was created by dissolving PbCl_2_ and MAI in a 1:3 ratio in DMF. The solution was then spin-coated onto substrates at 1,000 r.p.m. for 10 s followed by 5,000 r.p.m. for 20 s. Annealing was carried out by leaving the films at room temperature for 30 min followed by heating at 100 °C for an hour.

### Device fabrication

FTO-coated glass substrates (100 × 25 mm, 2.3 mm thick TEC15, Pilkington) were first etched with Zn power and aqueous hydrochloric acid (37%), and then cut into 25 × 25 mm pieces followed by cleaning sequentially in acetone, distilled water and IPA under sonication for 10 min during each washing cycle. A compact TiO_2_ layer was prepared by spin coating a solution comprised of 350 μl titanium isopropoxide (Aldrich), 35 μl hydrochloric acid (37%) and 5 ml anhydrous ethanol at 5,000 r.p.m. for 30 s. The TiO_2_ films were sintered at 160 °C for 5 min and then at 500 °C for 45 min. Next, a mesoporous-TiO_2_ film was deposited onto this using a solution of 20 nm particle transparent titania paste (18NR-T, Dyesol). The solution was spin-coated onto the substrates at 5,000 r.p.m. for 30 s. Once spun, the films were dried on a hotplate at 80 °C for 5 min, and then sintered at 500 °C for 45 min. The desired perovskite layer was prepared using a consecutive five-step spin program inside glovebox as previously described^40^. In instances where iodine salt layer were used, 1, 2, 6 and 10 mM solution of MAI with IPA were prepared respectively. The solution was then spin-coated onto the perovskite layer. The Spiro-OMeTAD hole conductor layer was spin-coated onto the perovskite films from a solution of 72.3 mg ml^−1^ 2,2′,7,7′-tetrakis-(N,N-di-p-methoxyphenylamine)9,9′-spirobifluorene (spiro-OMeTAD) powder in 1 ml anhydrous chlorobenzene. The spiro-OMeTAD solution contained additives including 17.5 μl lithium bis(trifluoromethane) sulfonimide lithium salt (Li-TFSl) and 28.8 μl 4-tert-butylpyridine (tBP). Finally, a 100 nm-thick gold contact was evaporated under vacuum (approximately 10^−6^ T at a rate of 0.2 nm s^−1^) with an active pixel area of 0.12 cm^2^.

### Current–voltage (JV) measurements

The JV characteristics were carried out using an AM1.5 simulated solar illumination (Oriel Instruments) and a Keithley 2400 source meter. Calibration was performed with a silicon photodiode before measurement. The scans were performed at a rate of 0.125 V s^−1^ for both forward scan (from short circuit open circuit) and backward scan (in the opposite direction). Cells were placed unmasked under continuous 1 sun illumination during aging and were masked during each scan to ensure the active area (0.12 cm^2^) is same for all measured devices.

### Superoxide measurements

A stock 31.7 μM solution of the HE probe was prepared by dissolving 10 mg in 10 ml of dry toluene; sonication was used to facilitate miscibility. Films were then added to 10 ml of 0.317 μM solution created from the stock solution. Photoluminescence spectra were recorded using an excitation wavelength of 520 nm and slit widths of 10 mm on a Horiba Yobin-Ybon Fluorolog-3 spectrofluorometer.

### Film coatings

A 0.01 M solution was prepared by dissolving the iodide salt (phenylethylammonium iodide, MAI or trimethylsulfonium iodide) in a 1:4 solvent mixture of IPA to chlorobenzene. One hundered microlitres of this solution was then dripped onto pre-deposited perovskite films with a 20 s loading time before spinning at 4,000 r.p.m. and annealing at 100 °C for 5 min. The chloride salt derivatives of the cations phenylethylammonium and methylammonium were prepared using the same protocol.

### Ageing conditions

Films were sealed in a controlled environment, where dry air was gassed through for the duration of the degradation and illumination was provided by a tungsten lamp equipped with a UV-blocking filter as previously reported^38^,^40^.

### Spectroscopy and microscopy

^1^H NMR was recorded on a 400 MHz Bruker spectrometer running and analysed using TopSpin software. Deuterated acetone was employed as the reference. UV–Vis was performed on a PerkinElmer UV/VIS Spectrometer Lambda 25. XRD patterns were measured on a PANalytical X'Pert Pro MRD diffractometer using Ni filtered Cu *K*_α_ radiation at 40 kV and 40 mA. SEM-EDX measurements were carried out on a JEOL 6400 scanning electron microscope operated at 20 kV. IGA measurements were conducted on a Mettler Toledo TGA spectrometer. For ToF-SIMS samples were fabricated onto clean glass substrates. The samples were then soaked under dry flux in the dark for an hour before the ToF-SIMS measurements were recorded. Data were obtained using an IONTOF ToF.SIMS-Qtac LEIS spectrometer employing an Argon sputter gun for oxygen ion detection.

### *Ab initio* calculations

DFT calculations were performed using the numeric atom-centred basis set all-electron code FHI-AIMS^71^,^72^. Tight basis sets were used with tier 2 basis functions for all species. Electronic exchange and correlation were modelled with the semi-local PBE exchange-correlation functional^73^. For the treatment of spin orbit coupling we used an atomic zeroth-order regular approximation (ZORA)^71^. Van der Waals forces were accounted for by applying a Tkatchenko–Sheffler electrodynamic screening scheme^74^. O_2_ absorption in the bulk was calculated using a 2 × 2 × 1 supercell (giving a tetragonal cell of 192 atoms). A gamma point offset grid at a density of 0.04 Å^−1^ was used for *k*-point sampling. Structures were relaxed with convergence criteria of 10^−4^ eV Å^−1^ for forces, 10^−5^ electrons for the electron density and 10^−7^ eV for the total energy. These settings ensured highly converged energies and equilibrium distances.

### Data availability

The data that support the findings of this study are available from the authors on reasonable request.

## Additional information

**How to cite this article:** Aristidou, N. *et al*. Fast oxygen diffusion and iodide defects mediate oxygen-induced degradation of perovskite solar cells. *Nat. Commun.* **8**, 15218 doi: 10.1038/ncomms15218 (2017).

**Publisher's note:** Springer Nature remains neutral with regard to jurisdictional claims in published maps and institutional affiliations.

## Supplementary Material

Supplementary Information Supplementary Figures, Supplementary Table and Supplementary Notes
