# Supplementary material for: Fast oxygen diffusion and iodide defects mediate oxygen-induced degradation of perovskite solar cells
Source: Nat Commun. 2017 May 11;8:15218. doi: 10.1038/ncomms15218 (PMC5437277; doi:10.1038/ncomms15218)
Supplement: Supplementary Information — Supplementary Figures, Supplementary Table and Supplementary Notes [file ncomms15218-s1.pdf]

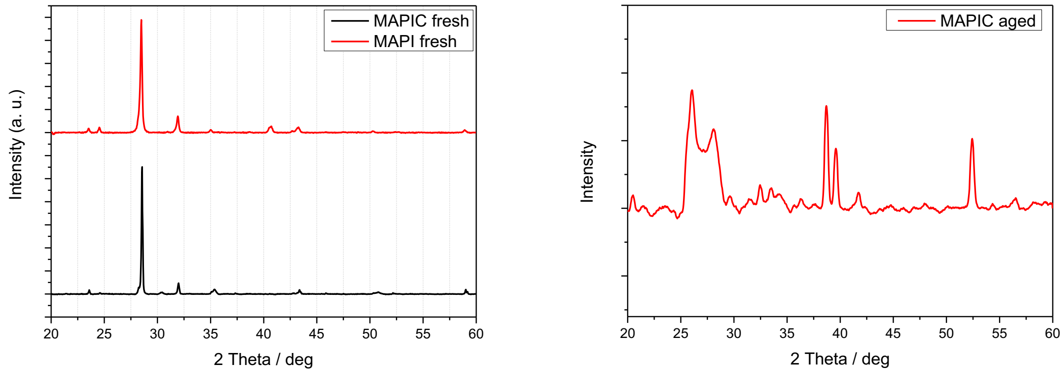

Supplementary Figure 1. XRD Patterns

Left: XRD patterns of $CH_3NH_3PbI_3$ and $CH_3NH_3PbI_3(Cl)$ films. Right: XRD pattern of a degraded $CH_3NH_3PbI_3(Cl)$ film.

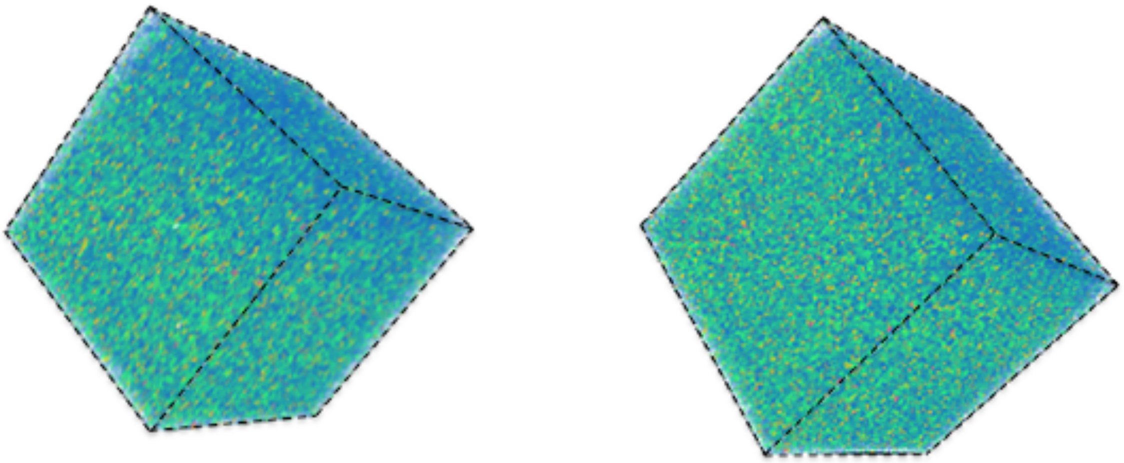

Supplementary Figure 2. 3D depth profiles for O_2 in Perovskite films

Left: ToF-SIMS 3D Oxygen ion plot of a MAPI film. Right: ToF-SIMS 3D Oxygen ion plot of a MAPIC film.

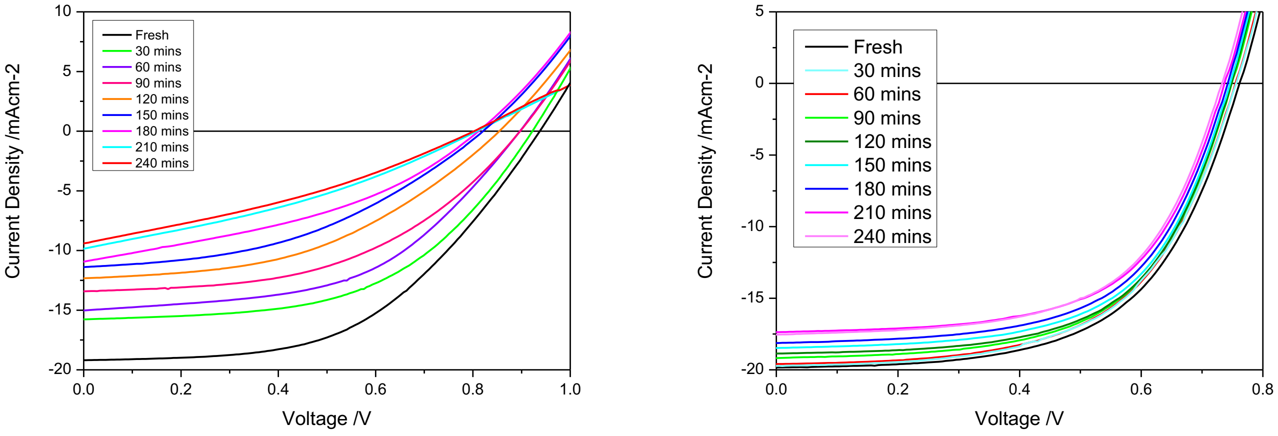

Supplementary Figure 3. Aging Solar cells under oxygen and light

J-V curves obtained for $CH_3NH_3PbI_3$ (left panel) and $CH_3NH_3PbI_3(Cl)$ (Right panel) devices employing an FTO/c-TiO2/mp-TiO2/Perovskite/Spiro-OMeTAD/Au architecture. Devices were degraded under one sun illumination and dry air-flow.

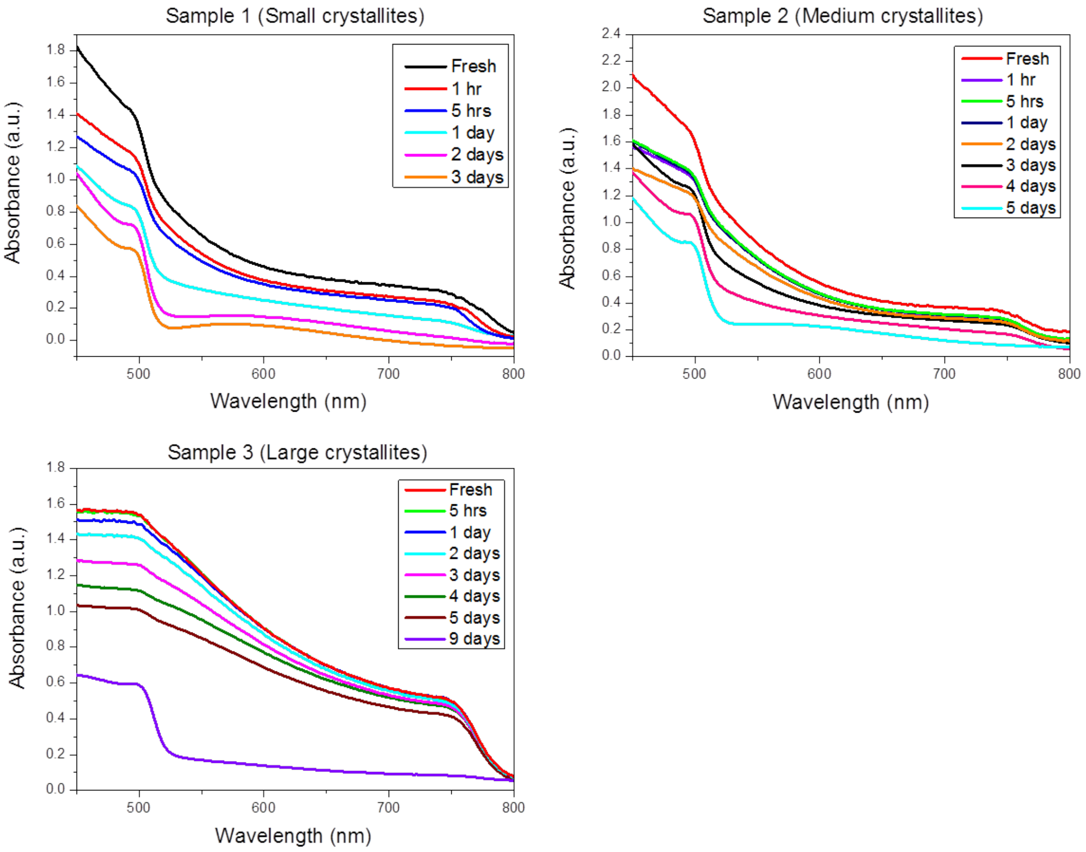

Supplementary Figure 4. Absorbance profiles of different crystallite sizes vs ageing time

Raw absorbance data collected for methylammonium iodide films with varying crystallite sizes Sample 1 (100 nm), Sample 2 (150 nm) and Sample 3 (250 nm) under dry air flow and illumination (25 mW/cm^2)

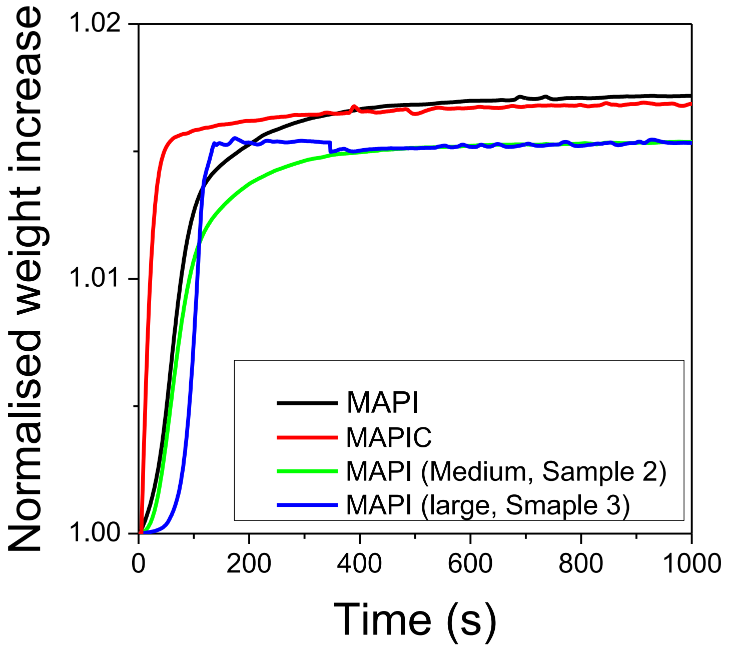

Supplementary Figure 5. Oxygen diffusion into perovskite films

IGA results for different perovskite films.

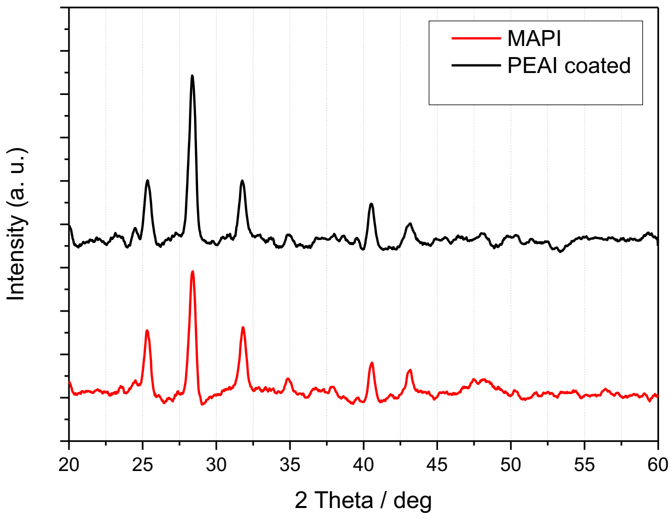

Supplementary Figure 6. Salt coating impact on XRD patterns

XRD patterns obtained for $CH_3NH_3PbI_3$ films coated with phenylethylammonium iodide. $CH_3NH_3PbI_3$ XRD pattern is shown as reference.

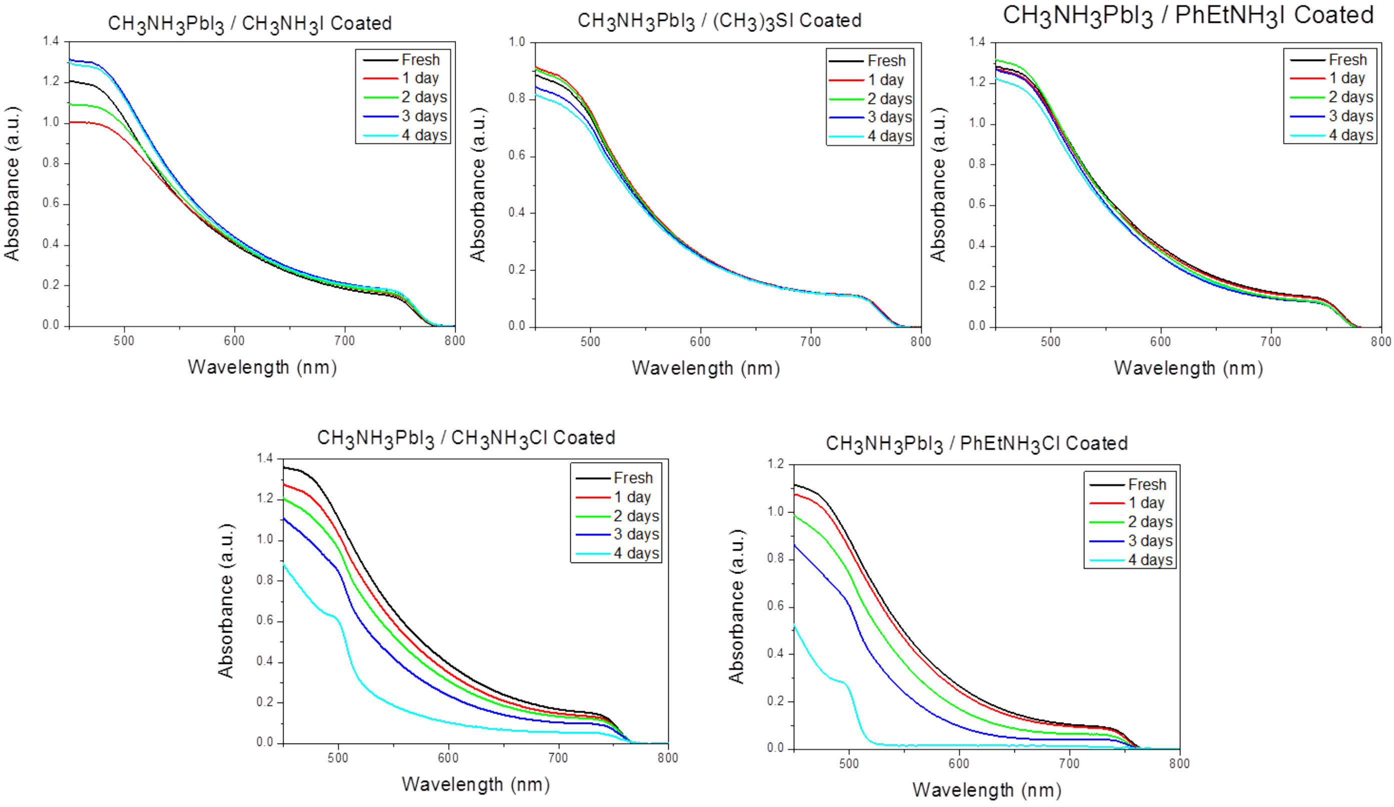

Supplementary Figure 7. Absorbance profiles of different salt treatments vs ageing time

Raw absorbance data collected methylammonium iodide films coated with methylammonium iodide (Top Left), trimethylsulfonium iodide (Top Middle), phenylethylammonium iodide (Top Right), methylammonium chloride (Bottom Left) and phenylethylammonium chloride (Bottom Right). Films were aged under dry air flow and illumination (25 mW/cm^2).

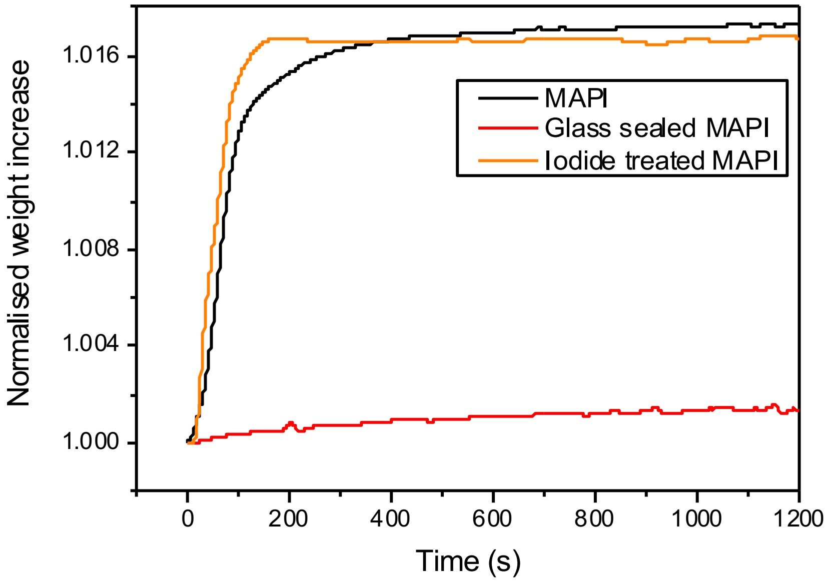

Supplementary Figure 8. Oxygen diffusion salt treatment vs blocking layer

IGA results for MAI coated and uncoated $MAPbI_3$ films. Also shown are IGA data for a MAPbI3 film encapsulated in glass.

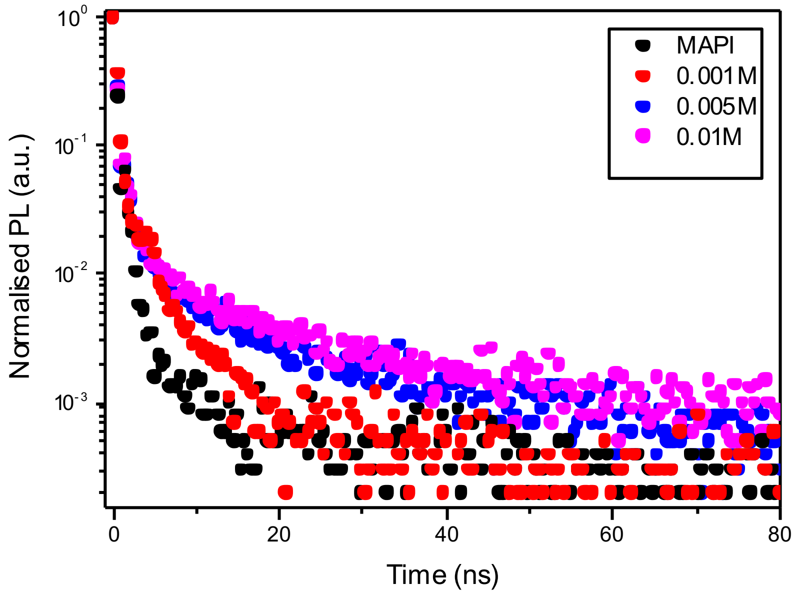

Supplementary Figure 9. Photoluminescence as a function of salt treatment concentration

Time resolved photoluminescence data as a function of concentration of MAI coating solution. Also shown are emission kinetics for an uncoated control sample (black decay).

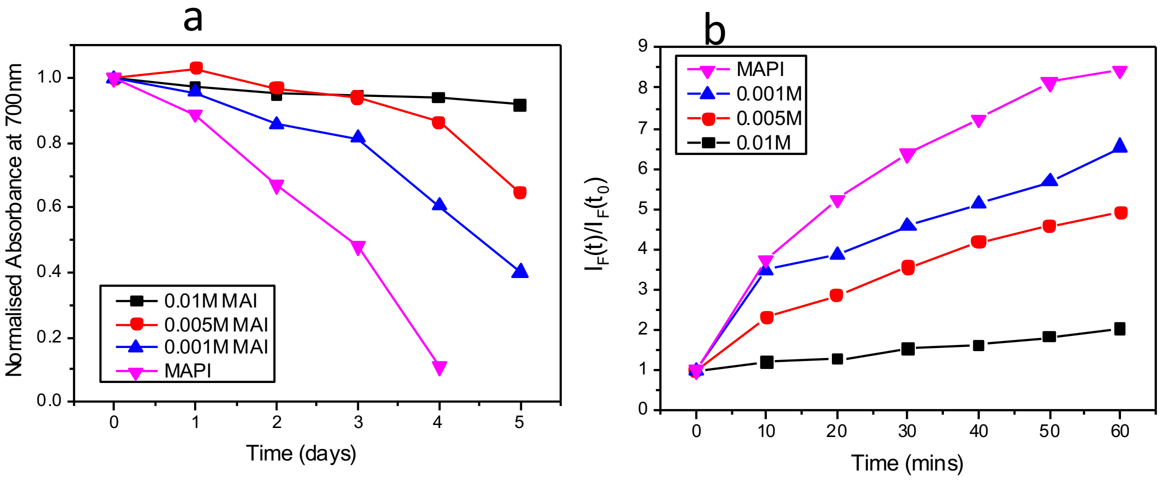

Supplementary Figure 10. Salt treatment concentration and stability enhancement

a) Normalised absorbance degradation plot indicating that increasing the concentration of MAI salt treatment leads to an enhanced stability (longer retention of absorption at 700 nm). b) Superoxide generation plot as a function of MAI concentration. Indicates that increasing superoxide is observed for reduced slat concentrations.

.

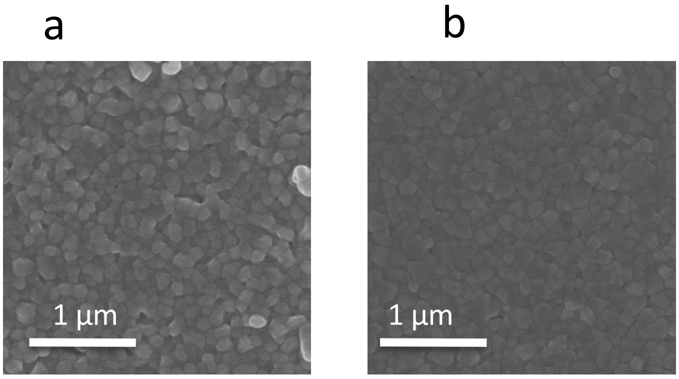

Supplementary Figure 11. Morphology of Salt treatment

Scanning electron microscopy studies for MAI coated (a) and uncoated (b) $MAPbI_3$ film.

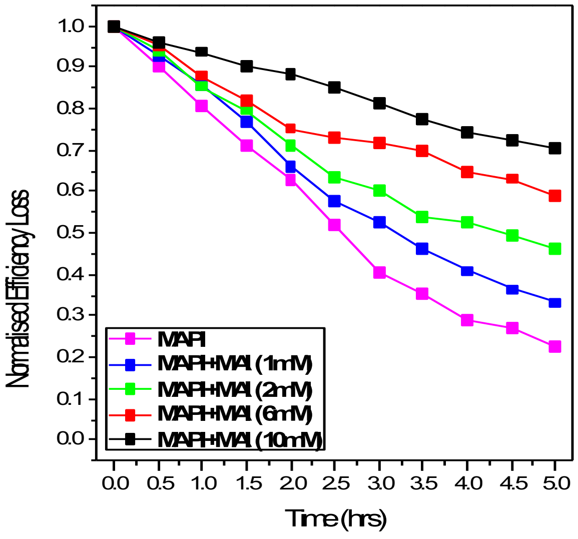

Supplementary Figure 12. Normalised PCE degradation as a function of salt concentration

Normalised PCE loss as a function of ageing time under 1 sun illumination with dry air flux, for MAPI films with no coating, 1mM, 2mM, 6mM and 10mM of methylammonium iodide salt coating.

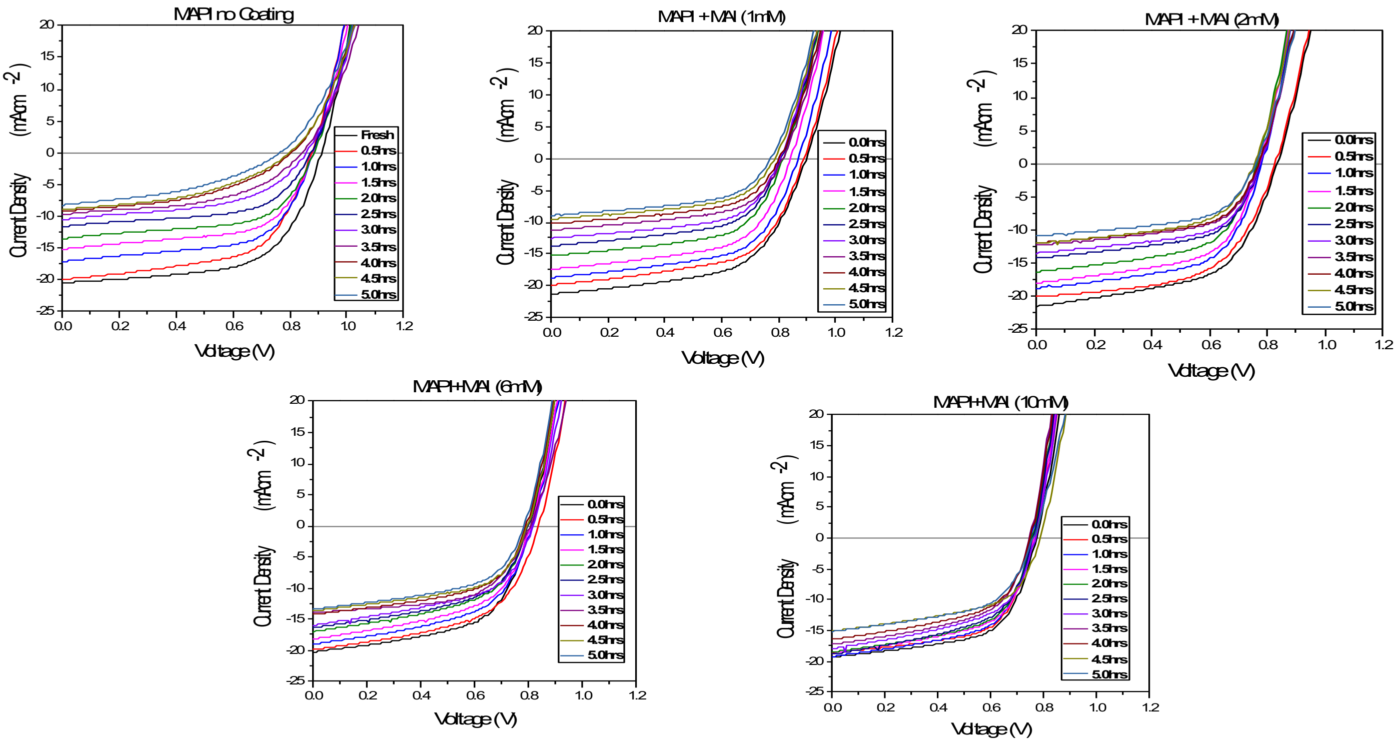

Supplementary Figure 13. J-V curves as a function of salt concentration and degradation

J-V curves obtained during degradation for devices with a MAPI active layer and for a MAPI active layer with methylammonium iodide treatment with concentrations of: 1mM, 2mM, 6mM and 10mM.

Supplementary Table 1. Photovoltaic parameters for devices based on MAI coated $MAPbI3$ perovskite films.

Device Type	Pixel Area (cm^2)	Voc (V)	Jsc (mA/cm^2)	FF	Efficiency (%)
MAPI (Ref.)	0.12	0.9123	20.481	0.6105	11.394
MAPI + MAI (1mM)	0.12	0.8977	21.286	0.5810	11.098
MAPI + MAI (2mM)	0.12	0.8339	21.512	0.5631	10.098
MAPI + MAI (6mM)	0.12	0.8069	20.277	0.5784	9.4560
MAPI + MAI (10mM)	0.12	0.7745	19.288	0.6039	9.0152

Supplementary note 1. Oxygen Diffusion Coefficient Calculation:

- Film area ~ 3×10^{-6} cm^2 ± 1×10^{1} cm^2
- We note the area involved relates to the depth of the film
- Time taken to saturate film ~ 300 s
- Therefore: $D = 3\times10^{-6} / 300 \sim 1\times10^{-8} \pm 1\times10^{1}$ cm^2 s-1
- The film area is calculated from the following dimensions:
  - width: 0.1cm
  - height (film thickness): 300 nm

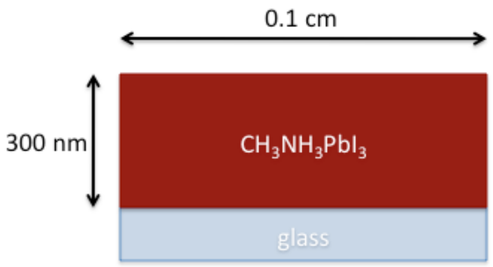

Supplementary note 2. Calculation of O_2^{-} formation energy:

The formation energy for superoxide insertion and reduction was computed using the equation;

$$\Delta E_{SF} = E^{C}(dMAPI + O_2) - E(O_2) - E^{C}(dMAPI)$$

Where;

$E^{C}(dMAPI + O_2)$ is the total energy of the defect-containing MAPI supercell with charge state C containing inserted and reduced O_2.

$E^{C}(dMAPI)$ is the total energy of the defect-containing MAPI supercell with charge state C prior to O_2 insertion.

$E(O_2)$ is the energy of an isolated O_2 molecule.

Notes:

- ΔE_{SF} contains contributions from both oxygen reduction at the site together with the energy change upon insertion of O_2 at the site.
- The charge state, C, of the host lattice is unchanged during the process – the sites are considered to be photo-reduced/oxidised prior to O_2 insertion.
- For possible defect sites on which to reduce O_2 we considered positively charged and neutral (ie photo-reduced) anion vacancies together with negatively charged (and photo-oxidised) cation vacancies. The defect free bulk was also considered in both a neutral state and with an excess photo-generated electron.
